# Collagen architecture in triple negative breast cancer

**DOI:** 10.1371/journal.pone.0324655

**Published:** 2025-05-21

**Authors:** Zoë Dinkel, Adam Baker, Alannah Akins, Kylie L. King, Matthew Harrington, Deborah Kunkel, Zhi Gao, Tong Ye, Heather Dunn

**Affiliations:** 1 Department of Bioengineering, Clemson University, Clemson, South Carolina, United States of America; 2 School of Mathematical and Statistical Sciences, Clemson University, Clemson, South Carolina, United States of America; University of Pennsylvania School of Engineering and Applied Science, UNITED STATES OF AMERICA

## Abstract

This study evaluated collagen properties in TNBC samples collected from different racial groups to determine the presence of variance in matrix architecture. African American (AA) breast cancer patients have a significantly higher mortality rate and nearly a three-fold increased prevalence of triple negative breast cancer (TNBC) when compared to Caucasian (C) patients. The extracellular matrix region surrounding tumors contains abundant collagen fibers, and these fibers undergo remodeling throughout cancer progression, promote metastasis, and impede treatment response. High mammographic density, commonly known as dense breast tissue, is hypothesized to be more prevalent in AA women and characterized by increased collagen deposition and associated with more aggressive cancers. The aim of this research was to investigate fibrillar collagen architecture in TNBC samples from AA and C patients using two-photon microscopy with second harmonic generation (SHG), an intrinsic optical signal produced by fibrillar collagen. Twenty tissue regions per tumor sample were randomly selected for SHG microscopy, and image processing was conducted using the Fiji macro TWOMBLI to quantify mesoscopic fibrillar morphological properties and nanoscopic fibrillar properties with the Forward-Backward SHG ratio. Compared to the images from C tumor samples, those from AA samples exhibited a significant increase in parameters including fiber area, total length, and number of endpoints and branchpoints, but had decreased lacunarity. Collagen microstructure, including fibril arrangement and packing density, did not significantly differ between the groups. These results illustrate that the TNBC samples analyzed from AA patients may have macrostructural collagen characteristics associated with aggressive phenotype tumor formation.

## 1. Introduction

One in eight women will develop invasive breast cancer (BC) during her lifetime, making this disease the most prevalent female cancer and the second leading cause of cancer death across women in the U.S. [1,2]. Triple negative breast cancer (TNBC) is one of the most aggressive BC subtypes due to the absence of ER, PR, and HER2 amplification. Accounting for 10–15% of all breast cancers, TNBC is associated with increased invasiveness, distant metastasis, recurrence, and poor prognosis [3]. When compared to Caucasian (C) women, African American (AA) women experience a 42% higher BC mortality rate, diagnosis at a younger age and with later stage disease, larger, more aggressive tumors, and a 2.7 times higher risk of a TNBC diagnosis [2,4–6]. These racial disparities have been associated with biological and socioeconomic factors, including health care access, income, obesity, and lack of participation in clinical trials [5,7].

The primary cause of BC mortality is the metastasis of the tumor to distant sites, which is influenced by the components of the tumor microenvironment including the extracellular matrix (ECM) [8]. Composed of water, proteins, and signaling molecules, the ECM is the dynamic, non-cellular component within tissues that provides mechanical support, influences immune responses, and regulates cell behavior [8,9]. The ECM is constantly undergoing tightly controlled remodeling involving deposition, degradation, or modification of its components, which influences tissue tension, induces migration highways, and elicits varied biochemical and biomechanical functions [9–11]. However, cancer promotes deregulated reorganization, stiffening, and compositional alterations of the ECM which influences tumor progression and treatment response [8,12]. The cancerous ECM promotes cell proliferation, differentiation, survival, invasion, and metastasis by forming a platform for cell adhesion and migration, conducting cell signaling, and undergoing structural changes that promote an aggressive phenotype, including the increased deposition of fibrillar collagen [8,11,12].

Collagen is the most abundant protein in the ECM, accounting for up to 30% of a human’s total protein content and providing strength and structural support throughout the body [9,11]. A collagen molecule is usually a triple-stranded helix composed of three polypeptide chains that can form a variety of supramolecular assemblies, including fibrils and networks [9,10]. In the mammary gland, the primary component of the interstitial ECM is type I collagen, a fibrillar collagen that provides structural support, while the basement membrane separating the epithelial cells from the stroma is composed of type IV collagen, a network-forming collagen [8,10,11]. During the epithelial invasion occurring in cancer, the cells interact with these collagens as they surpass the basement membrane and invade the interstitial ECM [11].

**Fig 1:**
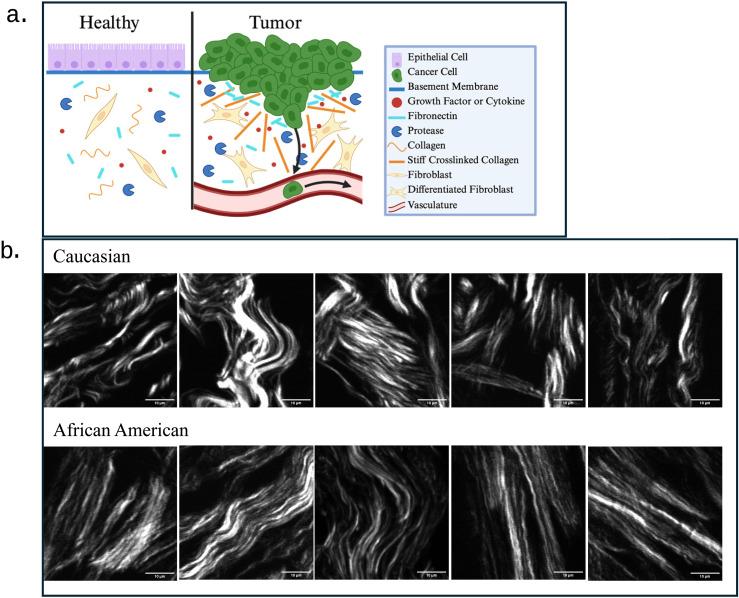
a) ECM components and collagen structure change with tumor progression, which influences cancer cell proliferation and metastasis. b) SHG visual representation of collagen patterns from ten unique patient samples where the upper row is from five C patient samples and the lower row is from five AA patient samples.

ECM architecture is influenced by collagen quantity, crosslinking, and proteolysis, which are all deregulated in cancer [11]. Throughout tumor progression, thin, relaxed, anisotropic, and curly collagen fibers progressively linearize, crosslink, thicken, orient perpendicular to the tumor border, and exert tension on epithelial cells, increasing the ECM stiffness and thereby supporting metastatic capabilities by forming highways to facilitate cancer cell migration towards the vasculature. Fig 1a provides a visual representation of changes in the ECM in healthy tissue compared to tumor tissue [8,11,13,14]. On the other hand, collagen can also constitute a physical barrier against invasion [8]. Increasing fibrillar collagen content, however, increases secretion of ECM degradation enzymes, thereby generating pathways for cancer cell migration [8]. Stiff and dense collagen networks may obstruct drug delivery by sequestering the drugs, raising the interstitial fluid pressure, or hindering large molecular weight drugs from diffusing towards the tumor [8,11]. High and low collagen concentrations have been associated with aggressive cancer [11]; therefore, examining collagen remodeling and architecture is essential to understand its effects on cancer.

Characterized by increased fibrillar collagen deposition, less adipose tissue, and greater proportions of stroma, high mammographic density (MD) is associated with a four to six times greater BC risk [15–17] and with more aggressive BC, including larger tumors, increased spread to nearby lymph nodes, and cancers that are high grade and stage, rapidly growing, and hormone-receptor negative [6,17–20]. Not only can dense tissue impede tumor detection, but increased MD may also promote cancer cell proliferation and growth [18]. High breast density has been associated with increased deposition, alignment, linearity, stiffness, organization, and thickness of collagen fibers [15,16,21,22], and collagen features may act as predictive markers for BC risk and aggression.

Second harmonic generation (SHG) is a powerful microscopy technique which yields images of collagen fibers, allowing for a label-free, highly specific signal to evaluate collagen morphology. Fig 1b provides SHG captured images from patient samples where the white patterns are the collagen fibers present in the ECM. The Forward-to-Backward (FB) ratio is a direct comparison between the transmitted (forward) and reflected (backward) SHG signal. In physical terms, FB indicates how tightly bound the collagen fibrils are within their larger bundle, which has been correlated to the total fiber diameter [23,24]. Because of its highly quantum nature, the direct racial implications of this quantitative image to patient health have not been investigated. However, previous SHG work has shown that collagen type III-type I ratio is proportional to SHG intensity, which has been linked with increased malignancy in BC [25,26]. The FB ratio has also been measured as an indicator of the presence of cancer in ovarian cancer [27].

When compared to C women, AA women generally have higher breast density [4,6,28–30], and this increased density has been linked to increased collagen present in breast tissue. Based on this information, the purpose of this study was to evaluate collagen variation as a possible contributing factor to the increased TNBC incidence rate and BC mortality rate. Collagen architecture influences cancer progression, but the impact of racial-ethnic groups on the molecular formation and structure of collagen in BC has not been extensively studied considering collagen features may act as a predictive marker for BC risk and aggressive properties. In order to evaluate the properties of collagen, we explored the direct correlation of FB signal intensity and nano- and mesoscopic morphological properties of the fiber to the difference between racial-ethnic groups in malignant BC.

## 2. Materials and methods

### 2.1. Patient specimens

This study was provided with ethics committee approval prior to commencing and approved by Clemson University Office of Research Compliance. The Institutional Review Board (IRB) is a federally mandated body established under the U.S. Department of Health and Human Services regulations for the Protection of Human Subjects policy 45 CFR 46. Its purpose is to protect the rights and welfare of human subjects recruited to participate in research activities conducted under the auspices of Clemson University, and this policy requires that all research involving human subjects be reviewed and approved by Clemson University’s IRB office prior to initiation of the research. This requirement applies to all human subjects’ research conducted by faculty, staff, and students, on- and off-campus for the project. Patient samples used for this study were obtained from the Greenville Health Cancer Institute Biorepository (Greenville, SC; IRBNet Study ID 1853278) and approved by the Clemson University Office of Research Compliance (IRB 2022–0688). Written informed consent for sample collection was obtained from all subjects and/or their legal guardian(s). The subjects signed and dated their consent with a healthcare professional present. We did not collect any data for this study from minors. Samples used for this study were obtained from patients and submitted to the biorepository between December 9, 2022 through November 17, 2023. The authors did not have access to any information that would identify participants during or after data collection. We collected a total of 27 formalin-fixed and paraffin-embedded (FFPE) tumor samples (17 C and 10 AA) from grade 3 TNBC patients. The C patient ages at collection ranged from 32–81 years (mean, 57.5 years; median, 60 years). The non-Hispanic AA samples were collected from Prisma Health between July and October 2022. The AA patient ages at collection ranged from 32–83 years (mean, 58.7 years; median, 51 years).

### 2.2. Second harmonic generation imaging

Ti-Sapphire femtosecond pulse laser (Tsunami, Spectra-Physics) was tuned to 830nm, and the output power was optimized by rotating a λ/2 waveplate in front of a polarizing beamsplitter. The sample was imaged with a 60X water immersion objective (NA = 1.0) and the 2D raster scan of the laser beam with a pair of galvanometers; the site of each image was approximately a 45.96 μm square and recorded as a 512x512 image, giving an approximate resolution of 0.13 µm/px with a dwell time of 6 microseconds. The forward path was first involved with an oil immersion condenser and then filtered with a 720 nm shortpass and a 414/46 nm bandpass Semrock filters. The backward path was filtered with a 740 nm and a 470 nm shortpass filters. The sample location was randomly chosen on each slide and imaged while the power was slowly increased until sufficient collagen signal was identified in the forward direction. Samples were histology grade and less than 5 microns in thickness. With the approximate slice thickness of 1.2 microns, we imaged a plane above and below the samples to ensure that adjacent layers were not contributing to the data. The gradual increase in power prevented hematoxylin and eosin (H&E) tissue regions from overheating. Random selections of tissue areas were imaged simultaneously in the forward and backward 20 times per sample, yielding a total of 540 images.

### 2.3. Power tuning

Power of the emitting light was first regulated by the pump, but because the samples used were stained with H&E, it was necessary to fine-tune the imaging beam to a lower power to avoid burning the sample. Power into the sample was optimized with an adjustable λ/2 waveplate placed in front of a polarizing beamsplitter and monitored with an OceanView Optics photodetector. The detector was placed in the beam’s path and the waveplate adjusted until power was minimized, then the detector was removed from the emission path. While using the live feed of the SHG image, the waveplate was then carefully adjusted to increase power until good resolution was achieved.

### 2.4. Image processing

Image processing was conducted using Fiji (v1.53t) which is an open source software package, and fiber analysis utilized The Workflow Of Matrix BioLogy Informatics (TWOMBLI) v1 macro [31]. AnaMorf, Ridge Detection, and OrientationJ were also included per the TWOMBLI recommended use guidelines. The macro first calculates the FB images from raw image stacks and then uses TWOMBLI to calculate the fiber mask image. The second phase creates fiber contouring and then FB ratio measurements along the drawn mask, measurements which were generated via a second lab-built macro to ease the processing load.

### 2.5. Fiber analysis TWOMBLI

**Fig 2:**
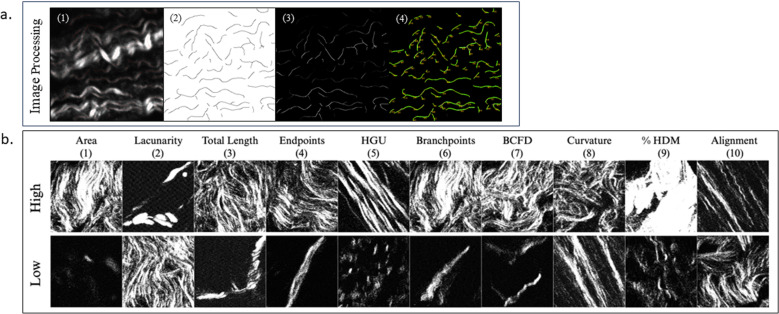
a) FB image processing featuring (1) FB map, (2) TWOMBLI mask, (3) FB-mask multiplied image, and (4) Ridge detection selection of contours. b) TWOMBLI Output Descriptions. (1) Area: cross-sectional area of all fibers, (2) Lacunarity: number and size of gaps in the ECM, (3) Total Length: sum length of all drawn fibers, (4) Endpoints: sum of fiber terminus points, (5) Hyphal Growth Unit: number of end points per unit length, (6) Branchpoints: number of fiber intersections, (7) Box-Counting Fractal Dimension: indicates self-similarity and space-filling properties, (8) Curvature: change in angle moving along individual fibers, (9) Percent High-Density Matrix: proportion of white pixels with respect to dark pixels, (10) Alignment: degree to which fibers are oriented in a similar direction.

TWOMBLI was developed for BC tissue to analyze fiber properties of histologic sections [31]. In the present study, the researcher was blinded to patient racial groups by using a random number generator to prevent bias during data analysis. The 20 images from each sample were divided based upon visual examination of length and curvature into two image sets, and the code was generated for each set of images. TWOMBLI analysis occurred in two phases. The first phase was the training phase, which utilized three random images and accepted user input that predicted fiber length, diameter, and curvature. During this study, all user inputs remained constant across all cycles of generated code except for curvature, which required attention due to its variation in output metrics as advised by Wershof et al. For all samples, contrast saturation was set to 0.35, line width was 15, minimum branch length was 15, maximum display high density matrix (HDM) was 200, minimum gap diameter was 17, and curvature ranged from 40 to 120 based upon the length of straight lines featured in the masks of the three random images. These training sets were verified and then applied to the remaining data set for processing with identical parameters. The second phase analyzed every image in the folder, created the fiber mask image, and calculated a comprehensive set of morphological data points (Fig 2a) [31]. These metrics were paired with the raw image and the drawn fiber mask.

### 2.6. Forward-to-backward second harmonic generation

Forward-to-Backward ratio was first calibrated on the system by placing a 50/50 beamsplitter in the illumination path, a mirror was placed on one perpendicular face and a 405 nm light emitting diode (LED) source on the opposite. To protect the photomultiplier tubes (PMT), the LED was not directly aimed at the beamsplitter but utilized black cardboard to reflect the light into the detector path. The measurements were taken from 100 frames and found to have an average FB of 1.079. Forward and Backward images were averaged from stacks of 20 frames at the exact location to improve contrast and thereby the fiber mask generated from TWOMBLI (Fig 2b). Light is circularly polarized when exciting the sample. Averaging the samples and windowing allowed for good fiber detection framing for this analysis. Those images’ ratios were used to make a quantitative FB ratio map. Once TWOMBLI generated the fiber masks, their pixel values were inverted and multiplied with the FB image to map the FB ratio contours. The only remaining data were TWOMBLI-identified fibers. These fibers within these masks were then selected using the built-in Fiji plugin ‘Ridge Detection,’ where each fiber was then processed as individual contouring ROIs; the drawn ROIs were then measured to generate Area (px^2^), Mean (μ), StdDev (σ), and contour length (px).

### 2.7. Statistical analysis

Twenty images of randomly selected regions were captured for each tumor sample, TWOMBLI evaluated 10 morphological parameters for each image, and the measurements for each tissue specimen were averaged (S1 Table). Utilizing the TWOMBLI-generated outputs, the average fiber length per sample was computed by dividing the total length by half of the sum of endpoints and branchpoints. Approximate fiber thickness was calculated by dividing the product of high-density matrix and field of view area by average fiber length [31]. Two-sample t-tests assuming unequal variances were performed through R Version 4.2.2 to evaluate whether the means of the morphological parameters differed significantly across racial groups. Statistical significance was determined using the Benjamini–Hochberg adjustment to control the false discovery rate (FDR) of 0.05. To assess sensitivity to normality assumptions, Mann-Whitney tests were also performed. Boxplots were created with Microsoft Excel Version 16.82. Correlation analysis was performed using the R package ‘corrplot’ [32].

A Principal Component Analysis (PCA) was completed to simplify the data set by reducing a set of correlated variables into a smaller set of uncorrelated variables by clustering similar measurements. The reduced set of variables, called “principal components”, represent indices combining several variables into one measurement. The PCA “scores” are transformations of the original data onto a new space defined by the principal components. Two-sample t-tests assuming equal variances were performed to evaluate whether the means of the principal component scores differed significantly across the groups.

Mean FB ratios were plotted on a boxplot through data analysis using JMP Pro Version 17. Exploratory tests were performed to assess whether features of the distribution of the mean FB ratios differed across the racial groups. For each sample, the following values were calculated to summarize the distribution of mean FB ratios: mean, median, standard deviation, and interquartile range (IQR) (S2 Table). These values were treated as independent measurements in two-sample t-tests with unequal variances and their means were compared across racial groups. Statistical significance was determined using the Benjamini–Hochberg correction with an FDR of 0.05.

## 3. Results

### 3.1. Mesoscopic morphology differs between African American and Caucasian TNBC tumors

**Fig 3:**
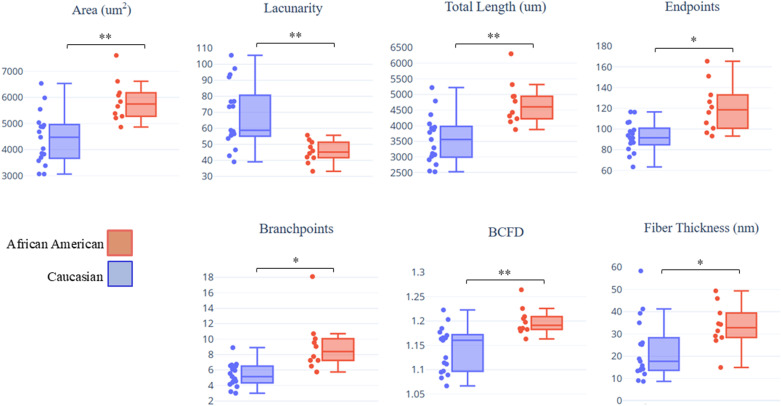
Boxplots of morphological parameters between racial groups. (a) Area increased in AA samples, (b) Lacunarity decreased in AA samples, (c) Total length increased in AA samples, (d) Endpoints increased in AA samples, (e) Branchpoints increased in AA samples, (f) Box-counting fractal dimension increased in AA samples, (g) Fiber thickness increased in AA samples, with *: significant at FDR = 0.05, **: significant at FDR = 0.01.

Among the 10 TWOMBLI parameters and two calculated parameters, seven were found to significantly differ between the racial groups based on a two-sample t-test (Table 1) (Fig 3). Area, total length, number of endpoints, box-counting fractal dimension (BCFD), number of branchpoints, and fiber thickness were significantly increased in AA samples when compared to C samples. Lacunarity, on the other hand, was significantly increased in C samples. There were no significant differences in hyphal growth unit (HGU), curvature, high density matrix (HDM), alignment, or average fiber length between the groups. Conclusions regarding statistical significance were similar when using the Mann-Whitney test.

**Table 1:** Summary of group means, standard deviations, t-statistics, and P-values.

	AA Mean (Std Dev)	C Mean (Std Dev)	T-Stat	Unadjusted P-value for T-Test	Unadjusted P-value for Mann-Whitney Test
Area	5872.4 (802.57)	4439.3 (1002.94)	4.08	0.0005**	0.0009
Lacunarity	45.4 (6.93)	68.1 (20.07)	-4.25	0.0003**	0.0006
Total Length	4724.3 (708.78)	3573.2 (772.60)	3.94	0.0008**	0.0007
Endpoints	120.9 (23.78)	92.4 (14.42)	3.44	0.0044*	0.0014
HGU	41.7 (5.76)	41.6 (7.31)	0.04	0.9710	0.7863
Branchpoints	9.2 (3.52)	5.3 (1.51)	3.30	0.0071*	0.0004
BCFD	1.2 (0.03)	1.1 (0.05)	4.08	0.0004**	0.0011
Curvature	29.3 (6.62)	25.4 (4.61)	1.67	0.1169	0.0929
HDM	0.1 (0.03)	0.1 (0.05)	2.22	0.0361	0.0459
Alignment	0.4 (0.07)	0.4 (0.08)	-0.18	0.8570	0.8245
Avg Fiber Length	73.8 (10.27)	73.4 (12.10)	0.10	0.9233	0.6392
Fiber Thickness	33.5 (9.88)	22.8 (13.59)	2.34	0.0283*	0.0175

^a^*: significant at FDR = 0.05, **: significant at FDR = 0.01.

### 3.2. TWOMBLI outputs are highly correlated

**Fig 4:**
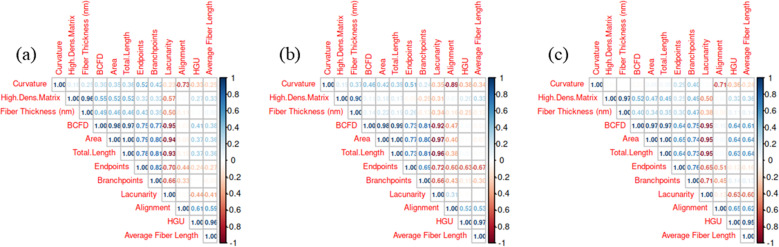
Correlation Plots Among TWOMBLI Outputs in (a) Combined samples, (b) AA samples, (c) C samples.

The correlations among the ten TWOMBLI outputs were plotted [32] (Fig 4). Area, BCFD, and total length were all highly positively correlated across both racial groups while lacunarity had a strong negative correlation with this group of variables; therefore, these variables all capture similar information. Branchpoints and endpoints also had a moderately strong positive correlation with area, BCFD, and length. Overall, the patterns of correlation were similar when calculated separately between the AA and C samples, though there was a moderately strong positive correlation of HGU and average fiber length with the group of variables containing area, BCFD, and length among the C samples, whereas these variables appeared unrelated in the AA group.

### 3.3. Parameters can be grouped to account for the variability

A PCA was completed since several TWOMBLI outputs were highly correlated and therefore captured similar information, determining that 78% of the total variability was accounted for by the first two principal components (PC1 and PC2), and 95% was accounted for by the first four principal components (Table 2). PC1 primarily captures the variability due to area, lacunarity, length, BCFD, and, to a strong but lesser degree, branchpoints and endpoints. PC2 primarily captures the variability due to HGU, alignment, average fiber length, and, to some degree, curvature. PC3 primarily captures variability due to HDM, fiber thickness, and, to some degree, curvature. The P-value for the test of equality of means for PC1 was 0.00025, for PC2 was 0.5115, and for PC3 and PC4 were similar large P-values. Therefore, it can be concluded that the variables associated with PC1 (area, lacunarity, length, BCFD, branchpoints, and endpoints) differ the most with respect to racial groups.

**Table 2:** Correlations of each parameter with PC1, PC2, and PC3.

	Correlation with PC1	Correlation with PC2	Correlation with PC3
Area	0.975	-0.069	0.185
Lacunarity	-0.935	0.186	-0.099
Total Length	0.975	-0.061	0.183
Endpoints	0.801	0.492	0.144
HGU	0.303	-0.894	0.142
Branchpoints	0.810	0.301	0.246
BCFD	0.969	-0.116	0.145
Curvature	0.418	0.641	-0.012
HDM	0.660	-0.182	-0.725
Alignment	-0.156	-0.859	0.002
Avg Fiber Length	0.298	-0.883	0.052
Fiber Thickness	0.625	0.045	-0.772

### 3.4. Collagen microstructure is comparable across African American and Caucasian TNBC samples

**Fig 5:**
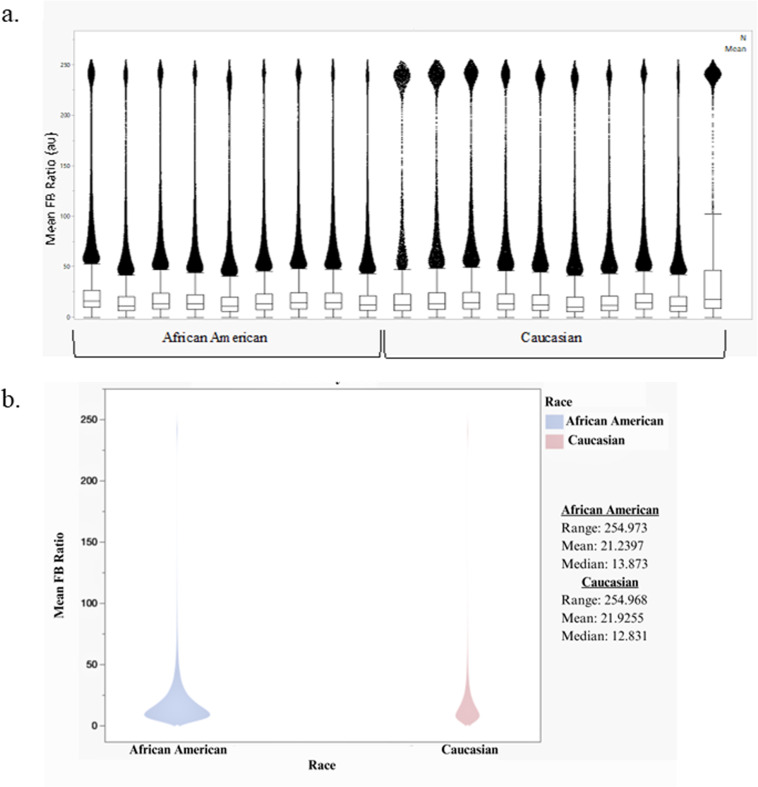
a.) Mean FB ratios by sample. b) Mean FB ratios by racial group.

Collagen fibril diameter, disorder, arrangement, and spacing within the larger fiber bundle influences forward SHG intensity, which can be quantified using the relative FB ratio [33]. Thicker fibers have been correlated with more densely packed fibrils and less empty space, resulting in increased forward-scattered light and therefore FB ratio [26]. Plotting generated mean FB ratios per sample indicates that most segments had a mean FB ratio between 0 and 50, and this pattern did not appear to differ much across samples (Fig 5). Additionally, the boxplots show many outlying observations falling into two clusters with one being centered around 50 and one being centered around 225. However, there appears to be substantial variability across samples in the volume of points in each cluster with C samples appearing to have a higher volume of points in the larger cluster than the AA samples.

Exploratory tests evaluating distribution features revealed that the mean, standard deviation, median, and IQR did not significantly differ between the groups, though there is some weak evidence of higher standard deviation in the C group than the AA group (Table 3). The number of measurements (N) obtained from each sample were also considered. These were classified as number measured (those falling between 0 and 255 and thus could produce an accurate measurement), number zeros (number of recorded zeros), number 255 (number of observations too saturated to measure), and total number (sum of the three preceding values). These values were all significantly higher for the AA group than the C group.

**Table 3:** Summary of mean FB ratio distribution across racial groups.

	AA Mean (Std Dev)	C Mean (Std Dev)	Difference (C Mean-AA Mean)	T-Stat	Unadjusted P-value for T-Test	Unadjusted P-value for Mann-Whitney Test
Mean	20.9 (2.0)	26.6 (13.01)	5.659	-1.4	0.2062	0.1823
Std Dev	28.4 (2.3)	41.1 (18.27)	12.712	-2.2	0.0561	0.0101
Median	13.6 (1.6)	13.4 (2.02)	-0.2333	0.3	0.7834	0.6607
IQR	15.2 (1.0)	17.6 (7.04)	2.3620	-1.0	0.3200	0.4967
N_Measured	218178.2 (119873.6)	70162.6 (47503)	-148,016	3.5	0.0058*	0.0002
N_Zeros	251356.9 (110885.2)	117668.2 (111813)	-133688.7	2.6	0.0183*	0.0041
N_255	144.9 (115.0)	42.0 (35.24)	-102.9	2.6	0.0290*	0.0199
Total N	469680.0 (220448.2)	187872.8 (153960)	-281807.2	3.2	0.0064*	0.0015

^a^ *: significant at FDR = 0.05.

## 4. Discussion

The most common BC subtype, ER and/or PR positive and HER2 negative, has a 5-year relative survival rate of 95% while TNBC has one of only 75% due to the lack of effective treatment [4]. Recorded rates for BC mortality and TNBC incidence are disproportionally higher in AA women than C women [5]. AA women also tend to have increased breast density [4,6,28–30], increasing the risk of BC formation and poor prognosis and establishing collagen variation as a possible contributing factor [34]. Collagen structure and organization undergo alterations during tumorigenesis that promote cancer progression, metastasis, and a more aggressive biology [8,14]. Few studies have explored the correlation between race, collagen, and BC outcomes. Angel et al. utilized genomic profiling to establish that fibrillar collagen genes differ between AA and C women and correlate to BC progression, as well as SHG microscopy to demonstrate that collagen fibrils from AA breast tumor samples are longer and less linear than C samples. The present study implements TWOMBLI and FB ratio to quantify collagen morphological properties from AA and C grade 3 TNBC samples.

A total of twelve parameters characterizing mesoscopic fiber morphology and general ECM patterning were evaluated with area, lacunarity, total length, BCFD, branchpoints, and endpoints differing the most between the racial groups. Area, total length, and BCFD were highly correlated and significantly increased while lacunarity decreased in AA tumor samples when compared to C samples, denoting greater collagen density. Increased stromal collagen has been shown to create an environment promoting cancer cell origination, proliferation, invasion, and metastasis [16,35]. Previous studies have shown that tumor cells migrate rapidly in regions of increased collagen deposition [11], which is also predictive of survival [36], and variance in collagen content may correlate to racial differences in BC progression and outcomes. Treatment responses and drug delivery have improved upon collagen reduction [11], which could be a potential therapeutic approach for AA women with TNBC.

The number of endpoints increased in the AA tumor samples, which may correlate to increased collagen degradation, a crucial process for the tumor growth-promoting abilities of fibrillar collagen [37]. Breaking down the collagen physical barrier and opening pathways for cancer cells, collagen proteolysis is critical for invasion and migration, as well as angiogenesis [8,11]. Cleaved type I collagen may also produce biologically active fragments promoting cancer cell migration and survival [11,38]. Though published research regarding the correlation of number of branchpoints to cancer progression is limited, the increase in branching among AA samples further exemplifies structural distinction across racial groups. No significant differences were found in curvature and alignment across the groups, but this analysis should be revisited in the future as linear, aligned fibers have been associated with aggressive cancers, cancer cell invasion, and worse disease-free survival [31,36].

The fiber area detected in the ECM is also associated with linearization or alignment of collagen fibers which can signal migration, invasion and epithelial mesenchymal transition (EMT) signaling [39–41]. Considering changes in collagen patterns are associated with ECM dysregulation and increased fiber area promotes cancer progression [42], the increased fiber parameters determined by this study for the AA group supports the increased incidence of aggressive tumors. Lacunarity refers to the fractal dimension of samples being analyzed [43] also described as two-dimensional spatial information [44]. This project detected significantly increased lacunarity from the C samples, which indicates increased spatial detection where collagen fibers were not present or not as abundant when compared to the AA group. The decreased levels of lacunarity in the AA samples reflected less two-dimensional space and increased collagen. The BCFD method is used to analyze complex patterns by reducing the dataset into a smaller boxed shape scale, and then estimating the dimensions [45]. This application has recently been used to quantify intestinal fibrosis [46], and this current study utilized BCFD values for the compactness of breast tissue. The BCFD was significantly increased in the AA group indicating increased tissue density. Increased fiber length of collagen architecture has been associated with elevated breast density and increased breast cancer risk [21], and this correlates to longer fibrillar collagen that creates elevated MD [22]. The fibers detected by this study indicated longer collagen patterns in the AA patients.

Fiber thickness increased in AA patients based on equations provided by Wershof et al. using TWOMBLI outputs. These thick collagen bundles in AA women can function as migration highways for tumor cells, promote BC invasion, and correlate to poor outcomes [13,47]. Thicker and more abundant collagen contributes to stromal stiffness [22,35], which results in an invasive, metastatic phenotype of mammary tumor cells, fosters cell migration to the vasculature, and upregulates a key molecule for angiogenesis, enabling the delivery of nutrients and oxygen to support tumor growth [11,48]. Our results have several possible implications. First, AA women may naturally have stiffer breast stroma than C women, correlating to an increased risk of BC and an aggressive phenotype as a stiff matrix is crucial for cancer emergence by inducing a tumor-like morphology and enhancing cellular proliferation [16,22,48]. Second, the stroma of AA women may undergo increased deregulated stiffening due to TNBC, which could contribute to cancer aggression and treatment resistance. Matrix stiffness influences the impact of chemotherapeutics on BC cells [49], so analyzing collagen patterns and recognizing stromal stiffness could lead to enhanced drug delivery.

Fiber diameter has been correlated with the FB ratio, which is highly sensitive to the spacing and parallel alignment of collagen fibrils bundled into the larger fiber; an increased ratio therefore correlates to an adjustment in one of these microstructural characteristics [23,33,50]. Though not significant, the FB ratio boxplot and standard deviation suggested that the C samples may have greater variability in microstructural properties than the AA samples, indicating that a notable number of their collagen fibers exhibited a remarkably larger FB ratio, which correlates to increased fiber diameter, fiber packing density, and potentially pathomechanical stiffness [48]. Though the FB ratio analysis does not support the previous conclusion drawn from TWOMBLI related to fiber thickness, the lack of variability in the mean FB ratio between patients and races does not interfere with the base hypothesis of this work. Where FB ratio indicates the organization and packing density of the fiber itself and is reflective of the means that the tissue creates new fibers, the arrangement of those fibers is driven by other factors. The pathomechanics of BC may be parsed here as we report no differences in the auto-assembly of the fibers but instead a measurable difference in the organization of new fibers. This may provide a key to understand which parts of the tissue are being directed and elucidate next steps we should take to understand the racial bias in the pathomechanical expression of BC. Further, the number of measured FB ratios, 0 FB ratios, 255 FB ratios, and total number of observed FB ratios were significantly larger from AA patients than C patients. This could either be a result of an increased number of collagen fibers per frame, supporting the previous claims from the TWOMBLI data, or a result of randomization.

The present study demonstrates that AA TNBC samples macroscopically differ from C samples and exhibit collagen patterns generally associated with facilitating BC metastasis, therefore developing a treatment that targets this unique collagen arrangement may help suppress cancer progression and overcome the less favorable outcomes these women face. Fully understanding the ways by which collagen structure may be a contributing factor to aggressive cancer could improve diagnostic measures and treatment strategies. Past studies have identified collagen as a possible therapeutic target to inhibit tumor growth and impede metastasis by preventing linearization, disrupting alignment, promoting degradation, or inhibiting cross-linking of fibers [13,51]. By establishing how exactly collagen structure differs between racial groups, an improved treatment can be developed specifically for AA women. Collagen has a strong predictive value in monitoring cancer progression and outcomes [36], so incorporating an examination of fibrillar collagen architecture into BC screening techniques could become a prognostic tool and help indicate a predisposition to aggressive cancer.

This study was conducted despite several limitations. The sample size was small due to limited availability of AA TNBC biopsy tissue. Future studies should include a larger cohort of patients for a more robust sample size, incorporate other racial groups, and evaluate nontumorigenic samples to assess the source of matrix variance as the collagen differences could indicate variance among the original healthy tissue or be a result of the cancer’s influence on the tumor microenvironment. Analyzing healthy breast tissue from AA and C women would therefore provide further insight into these racial differences.

## 5. Conclusions

Collagen within the ECM significantly influences tumorigenesis, metastasis, and survival and may be a contributing factor to aggressive cancer. Analyzing SHG images through TWOMBLI and FB ratio techniques is an effective method to investigate both macrostructural and microstructural properties of fibrillar collagen. TNBC samples from AA women are structurally different from that of C samples and exhibit a collagen morphology known to promote cancer progression, demonstrating the need for a racially informed analysis and treatment. Collagen has been recognized for its strong predictive value, so investigating collagen composition may enhance cancer monitoring and improve treatment strategies by directly targeting these fibers. Aiming to overcome the differences in TNBC outcomes, this study seeks to evaluate the contribution of collagen to BC racial incidence.

## Supporting information

S1 File Supplemental document. (DOCX)
