# Supplementary material for: Collagen architecture in triple negative breast cancer
Source: PLoS One. 2025 May 21;20(5):e0324655. doi: 10.1371/journal.pone.0324655 (PMC12094767; doi:10.1371/journal.pone.0324655)
Supplement: S1 File — (DOCX) [file pone.0324655.s001.docx]

Collagen architecture in triple negative breast cancer: supplemental document

**Table S1: Summary of averaged TWOMBLI parameters per sample.**

| **Race** | **Sample** | **Area (μm^2^)** | **Lacunarity** | **Total Length (μm)** | **End points** | **HGU (μm)** | **Branch points** | **BCFD** | **Curvature** | **% HDM** | **Alignment** | **Average Fiber Length (μm)** | **Fiber Thickness (nm)** |
| --- | --- | --- | --- | --- | --- | --- | --- | --- | --- | --- | --- | --- | --- |
| C | CU2 | 3386 | 92.00 | 2753 | 89.05 | 33.11 | 4.4 | 1.0973 | 27.4571 | 0.0441 | 0.2975 | 58.9149 | 15.7936 |
|  | CU3 | 5037 | 55.57 | 4058 | 106.25 | 40.01 | 6.5 | 1.1706 | 24.2005 | 0.1987 | 0.3729 | 71.9787 | 58.3114 |
|  | CU4 | 3858 | 76.64 | 3109 | 93.80 | 35.13 | 6.8 | 1.1249 | 25.3106 | 0.0352 | 0.3225 | 61.8319 | 12.0251 |
|  | CU5 | 4937 | 56.89 | 3953 | 91.50 | 53.52 | 5.6 | 1.1658 | 21.0137 | 0.0726 | 0.4347 | 81.4549 | 18.8269 |
|  | CU6 | 4868 | 58.05 | 3786 | 106.75 | 39.28 | 6.0 | 1.1772 | 27.2885 | 0.0454 | 0.3940 | 67.1872 | 14.2735 |
|  | CU7 | 3700 | 73.48 | 3019 | 97.65 | 32.99 | 4.6 | 1.0952 | 30.6018 | 0.0243 | 0.3265 | 59.0783 | 8.6884 |
|  | CU9 | 6537 | 39.08 | 5220 | 116.55 | 45.71 | 8.9 | 1.2228 | 35.5830 | 0.1027 | 0.1979 | 83.2212 | 26.0673 |
|  | CU12 | 4037 | 73.73 | 3292 | 99.10 | 37.49 | 3.9 | 1.1143 | 22.2120 | 0.0275 | 0.3213 | 63.9553 | 9.0662 |
|  | CU13 | 4683 | 56.54 | 3874 | 86.20 | 48.69 | 5.2 | 1.1654 | 24.7661 | 0.1406 | 0.4472 | 84.8254 | 35.0122 |
|  | CU15 | 4477 | 58.74 | 3560 | 87.00 | 44.12 | 6.6 | 1.1605 | 23.4352 | 0.1485 | 0.3761 | 76.0994 | 41.2197 |
|  | CU16 | 3065 | 105.58 | 2549 | 76.35 | 36.32 | 3.2 | 1.0894 | 21.2323 | 0.0538 | 0.3745 | 64.0867 | 17.7327 |
|  | CU17 | 5546 | 46.56 | 4354 | 91.30 | 51.69 | 4.9 | 1.1849 | 14.5170 | 0.0588 | 0.5383 | 90.5679 | 13.7140 |
|  | CU18 | 3827 | 76.84 | 3072 | 63.40 | 50.21 | 3.0 | 1.1117 | 24.0599 | 0.0588 | 0.4907 | 92.5331 | 13.4113 |
|  | CU20 | 5983 | 42.86 | 4790 | 95.55 | 52.12 | 6.1 | 1.2032 | 26.8304 | 0.1126 | 0.4333 | 94.2489 | 25.2249 |
|  | CU50 | 3069 | 97.30 | 2526 | 73.00 | 36.02 | 4.2 | 1.0669 | 26.9812 | 0.0789 | 0.3439 | 65.4763 | 25.4377 |
|  | CU51 | 3576 | 93.55 | 2910 | 80.75 | 35.49 | 4.6 | 1.0833 | 30.3778 | 0.0461 | 0.3630 | 68.2274 | 14.2571 |
|  | CU52 | 4881 | 53.65 | 3920 | 116.30 | 34.94 | 6.7 | 1.1634 | 25.3672 | 0.1186 | 0.2908 | 63.7690 | 39.2857 |
| AA | CU21 | 7609 | 33.19 | 6302 | 151.05 | 44.65 | 18.1 | 1.2643 | 32.7786 | 0.1027 | 0.3198 | 74.5143 | 29.0991 |
|  | CU22 | 5212 | 51.34 | 4127 | 100.75 | 42.46 | 7.3 | 1.1847 | 29.5306 | 0.0979 | 0.3590 | 76.4278 | 27.0439 |
|  | CU23 | 6080 | 41.64 | 4935 | 121.15 | 41.95 | 9.6 | 1.2051 | 27.7318 | 0.1765 | 0.3540 | 75.5111 | 49.3595 |
|  | CU24 | 5385 | 48.32 | 4311 | 96.45 | 47.81 | 5.8 | 1.1829 | 20.8380 | 0.1135 | 0.4835 | 84.3718 | 28.4157 |
|  | CU27 | 5658 | 45.93 | 4425 | 126.35 | 35.89 | 7.8 | 1.1796 | 27.3432 | 0.1435 | 0.4318 | 65.9978 | 45.9285 |
|  | CU29 | 6617 | 38.34 | 5317 | 165.50 | 33.79 | 10.7 | 1.2258 | 31.1890 | 0.0980 | 0.3253 | 60.3519 | 34.2826 |
|  | CU31 | 5279 | 52.90 | 4224 | 116.05 | 38.98 | 7.3 | 1.1852 | 37.1373 | 0.1278 | 0.3018 | 68.5126 | 39.4022 |
|  | CU45 | 6178 | 42.06 | 4942 | 132.95 | 38.31 | 9.1 | 1.2092 | 41.1254 | 0.1141 | 0.2391 | 69.6028 | 34.6273 |
|  | CU46 | 5841 | 44.35 | 4784 | 93.20 | 53.23 | 6.5 | 1.1977 | 25.5726 | 0.1426 | 0.4496 | 95.9609 | 31.3896 |
|  | CU47 | 4865 | 55.70 | 3877 | 106.00 | 39.65 | 10.1 | 1.1633 | 20.0475 | 0.0473 | 0.4004 | 66.8091 | 14.9550 |
| P-value | | **0.0005** | **0.0003** | **0.0008** | **0.0044** | 0.971 | **0.0071** | **0.0004** | 0.1169 | **0.0361** | 0.8570 | 0.9233 | **0.0283** |

**Table S2: Summary of FB ratio distribution per sample.**

| Race | Sample | N Rows | Mean | Std Dev | Median | Interquartile Range |
| --- | --- | --- | --- | --- | --- | --- |
| C | CU2 | 22321 | 26.995 | 48.622 | 12.555 | 16.264 |
|  | CU3 | 40903 | 26.402 | 43.903 | 14.150 | 16.216 |
|  | CU4 | 62986 | 25.687 | 40.442 | 14.643 | 16.501 |
|  | CU5 | 65961 | 22.980 | 36.102 | 13.336 | 15.412 |
|  | CU6 | 73086 | 22.008 | 35.139 | 12.750 | 15.094 |
|  | CU7 | 58448 | 19.532 | 33.350 | 10.713 | 14.388 |
|  | CU18 | 70481 | 19.708 | 29.665 | 11.880 | 14.762 |
|  | CU20 | 131259 | 21.847 | 30.157 | 14.261 | 15.006 |
|  | CU51 | 167378 | 17.982 | 24.769 | 11.449 | 14.431 |
|  | CU52 | 8803 | 62.575 | 89.069 | 17.797 | 37.458 |
| AA | CU21 | 172272 | 24.986 | 32.803 | 16.538 | 17.162 |
|  | CU22 | 100795 | 18.853 | 27.700 | 11.791 | 14.216 |
|  | CU23 | 188976 | 22.056 | 30.229 | 14.225 | 15.440 |
|  | CU24 | 167899 | 20.335 | 26.990 | 13.555 | 14.540 |
|  | CU27 | 152608 | 18.712 | 29.540 | 11.295 | 13.838 |
|  | CU31 | 180249 | 20.379 | 26.084 | 13.592 | 15.253 |
|  | CU45 | 513953 | 22.257 | 29.763 | 14.662 | 15.818 |
|  | CU46 | 267867 | 21.528 | 27.049 | 14.303 | 15.768 |
|  | CU47 | 218985 | 19.109 | 25.532 | 12.318 | 14.685 |
